# *In vitro* effects of undifferentiated callus extracts from *Plantago major* L, *Rhodiola rosea* L and *Silybum marianum* L in normal and malignant human skin cells

**DOI:** 10.1016/j.heliyon.2023.e16480

**Published:** 2023-05-24

**Authors:** Anette Gjörloff Wingren, Riyam Ziyad Faik, Anna Holefors, Edina Filecovic, Anna Gustafsson

**Affiliations:** a Department of Biomedical Science, Faculty of Health and Society, Malmö University, Malmö, Sweden; b Biofilms – Research Center for Biointerfaces, Malmö University, Malmö, Sweden; c In Vitro Plant-Tech AB, Geijersg 4B, 21618 Limhamn, Sweden

**Keywords:** Anti-inflammatory, Antioxidant, Flavonoid, HPTLC, Interleukin-6, Keratinocytes, *Plantago major* L, Trolox, Undifferentiated callus extracts

## Abstract

**Background and objectives:**

The occurrence of non-melanoma and melanoma skin cancers is currently increasing rapidly with one in every three cancers diagnosed as a skin cancer. A useful strategy to control the progression of skin cancer could be the use of plant flavonoids that suppress pro-inflammatory cytokines involved in tumor initiation and progression. In this study, the anti-inflammatory and antioxidant activity of undifferentiated callus extracts from *Plantago major* L, *Silybum marianum* L and *Rhodiola rosea* L was investigated both in normal and malignant skin cells.

**Methods:**

Antioxidant activity of the extracts was analyzed by using the Trolox Equivalent Antioxidant Capacity (TEAC) assay. High-Performance Thin-Layer Chromatography (HPTLC) was performed to demonstrate the phytochemical profile, and the total flavonoid content was analyzed with an aluminum chloride colorimetric method. The anti-inflammatory effect was investigated by cell treatments using the plant extracts. Thereafter, the possible suppression of induced IL-6 response was measured from the cultured skin cancer cell lines A2058 and A431, and normal primary keratinocytes with Enzyme-Linked Immunosorbent Assay (ELISA).

**Results:**

The HPTLC analysis assessed that the extracts contained a complex phytochemical profile that was rich in phenolic and flavonoid compounds. Dose response assays showed that concentrations between 15 and 125 μg/mL of all three plant extracts could be used to investigate an effect on the IL-6 production. The *S. marianum* extract had the most pronounced anti-inflammatory effect, which significantly inhibited induced IL-6 production in both normal keratinocytes and skin cells derived from epidermal carcinoma. The extract from *S. marianum* also had the highest flavonoid content and showed the highest antioxidant activity of the three extracts tested.

**Conclusion:**

All in all, we have confirmed that undifferentiated callus extracts of *S. marianum* possess properties such as antioxidant and anti-inflammatory activities in both normal and malignant keratinocytes, and thus could be a promising agent controlling the pro-inflammatory IL-6 production.

## 1 Introduction

Skin cancer, a disease caused by abnormal and uncontrolled growth of skin cells, is the manifestation of a series of genetic and epigenetic events, and it represents the most common form of malignancy in Caucasians [1,2]. Three major types of skin cancer are defined according to the affected layer: two non-melanoma types, basal cell carcinoma (BCC) and squamous cell carcinoma (SCC); and a third type, melanoma [3]. BCC and SCC are the most common types and rarely cause death, while the cutaneous melanoma starting in the pigment-producing melanocytes is the most aggressive type. Ultraviolet B (UVB) radiation from the sun is the most prominent factor for initiation of skin carcinogenesis by causing DNA damage, oxidative stress and inflammation [4]. UVB-irradiation can initiate intrinsic apoptosis through death receptor activation and through direct mechanisms such as DNA damage and oxidative stress. If the DNA damage caused by the resulting adducts is not repaired, a UV radiation specific permanent mutation is introduced into the genome. As a response to damage in the skin, interferon gamma (IFN-γ) is released by infiltrating T cells [5]. Cytokines released by T cells are among the strongest signals for chemokine expression in keratinocytes, and studies have shown that inflammatory skin displays intrinsic abnormalities in its response to pro-inflammatory factors. During the last decade, a growing number of experimental and epidemiologic studies have suggested a strong correlation between chronic inflammation and cancer [[6], [7], [8]]. Considering that chronic inflammation facilitates tumor initiation and plays a role in promotion/progression and tumor metastasis, the inflammatory pathways are attractive targets for skin cancer prevention. The inflammatory microenvironment is associated with pro-inflammatory cytokines produced by immune cells. Among these cytokines, the function of interleukin-6 (IL-6) as a master regulator of tumor-associated inflammation and tumorigenesis is well established [9].

Flavonoids, a class of structurally diverse polyphenolic compounds, have been shown to have various attractive features, including anti-inflammatory, anti-diabetic, anti-cancer, anti-aging, cardio-protective and neuro-protective effects [[10], [11], [12]]. With this in mind, the use of flavonoids, *i.e*., compounds that suppress inflammation, could be a useful strategy to control the development and progression of skin cancer. Flavonoids are metabolites located in the vacuoles of plants and can be extracted from all parts of the plant, such as the seeds, leaves, flowers and roots [13]. Higher plants have evolved to produce a wide range of phytochemicals to adapt to physiological changes and respond to various stress signals [14]. Depending on cell type, maturation grade and signal from the surrounding environment, different metabolites will be produced. The phytochemicals protect the plant from various detrimental extrinsic assaults, *e.g*., reactive oxygen and nitrogen species, UV-radiation, pathogens and parasites. Flavonoids have been reported to induce apoptosis in cancer cells, while not in normal cells [15], and have also been reported to act as kinase inhibitors, modulating cell signaling in skin cancer cell proliferation and apoptosis [16,17]. Inhibition of cell signaling through activator protein-1 (AP-1) and nuclear factor kappa B (NF-κB) activation is a central pathway that mediates anti-inflammatory effects of several flavonoids [18,19].

Even though the use of phytochemicals in many skin cancer cell lines and animal models have provided promising results regarding skin cancer intervention [20], the precise molecular mechanisms responsible have not yet been fully elucidated. In the present study, three plant samples were chosen, *i.e*., undifferentiated callus extracts from *Plantago major* L (greater plantain), *Silybum marianum* L (milk thistle) and *Rhodiola rosea* L (roseroot). Plant cell cultivation enables propagation of different cell types and organs, where depending on maturation grade and surrounding signals different metabolites will be used. In the current study undifferentiated callus cells were used to further elucidate the metabolic composition and activity in cells. We have chosen to target undifferentiated callus cells, since we were interested in their overall chemical profiles and activity when studied *in vitro*. The plants selected are all rich in flavonoids and are widely used in folk medicine around the world; and among other health benefits, they have been reported to possess anti-inflammatory and anti-cancer properties [[21], [22], [23], [24], [25], [26], [27]]. Silibinin, which is the main active component in *S. marianum*, is well investigated [28], while the other plant extracts in this study have been less studied in human skin cancer cell lines.

In the present study, the anti-inflammatory effect of extracts from undifferentiated callus cell lines from *P. major*, *R. rosea* and *S. marianum* extracts was investigated. Overall phytochemical content, total flavonoid content and antioxidant activity of the three extracts were analyzed. Normal epidermal keratinocytes from human skin together with human malignant skin cells derived from epidermal carcinoma (A431) and melanoma (A2058) were treated with the extracts, showing that undifferentiated callus extracts of *S. marianum* possess anti-inflammatory effects in both normal and malignant keratinocytes.

## 2 Materials and methods

### 2.1 Plant callus cell lines

Undifferentiated *Rhodiola rosea* L. (roseroot), *Silybum marianum* L. (milk thistle) and *Plantago major* L. (greater plantain) callus cell lines, previously developed by researchers at In-vitro Plant-tech (Malmö, Sweden), were used in the current study. Suspension cultures were elicited with RRE1, SME2 and PME2, harvested, filtrated, dried in a drying cabinet at 35 ^°^C and grinded to a fine powder.

#### 2.1.1 Extraction of bioactive substances

Bioactive substances were extracted using a multistep extraction process: 70%, 50% ethanol and water, to obtain extracts with a broad range of active substances with different chemical and polar properties. Biomass:solvent ratios of 1:20 were used. Extraction was performed using an ultrasonic water bath, 3 × 10 min cycles, followed by removal of supernatant by filtration. Prior to usage, the extracts were dried and resuspended in dimethyl sulfoxide (DMSO) to a concentration of 100 mg dry extract/mL DMSO. DMSO was used as a vehicle control in all experiments.

### 2.2 High-Performance Thin-Layer Chromatography (HPTLC)

For HPTLC analysis, 20 × 10 cm silica gel 60 F250 plates of 0.2 mm thickness were used (VWR, Gothenburg, Sweden). The plates were prewashed with methanol, and 10 μL of samples from extracts with a concentration of 10 mg/mL were applied using Automatic TLC Sampler 4 (CAMAG, Muttenz, Switzerland). The plates were developed with mobile phase containing ethyl acetate: methanol: water: formic acid, 30:5.1:3.9:0.8 (v/v/v/v), and derivatized using sulphuric acid for general visualization of a wide range of metabolites, and NP-PEG (natural products-polyethylene glycol) derivatization to visualize phenolic and flavonoids [29]. The plates were visualized under white light and 366 nm using the TLC Visualizer 2 (CAMAG, Muttenz, Switzerland) and scanned at 200–400 nm using the TLC Scanner 3 (CAMAG, Muttenz, Switzerland).

### 2.3 Determination of total flavonoid content

The aluminum chloride colorimetric method was modified from the procedure reported by Pekal and Pyrzynska [30]. Quercetin was chosen as a reference compound and dissolved in 100% ethanol to 1 mg/mL and then diluted to 12.5, 25, 50, 75, 100 and 150 μg/mL. Plant extracts were diluted in 100% ethanol to 0.625, 1.25 and 2.5 mg/mL. The diluted plant extracts and standard solutions (100 μL) were separately mixed with 300 μL of 100% ethanol, 20 μL of 10% aluminum chloride and 20 μL of 1 M potassium acetate. After incubation at room temperature for 30 min protected from light, the absorbance of the reaction mixture was measured at 415 nm. The amount of 10% aluminum chloride was substituted by the same amount of distilled water in the blank. The total amount of flavonoids in each extract was calculated from the linear standard curve and are expressed as mg quercetin equivalents (QE) per g dry extract.

### 2.4 Determination of antioxidant activity

Antioxidant activity was analyzed by using the Trolox Equivalent Antioxidant Capacity (TEAC) assay [31]. Trolox, (6-hydroxy-2,5,7,8-tetramethylchroman-2-carboxylic acid, Sigma Aldrich), a water-soluble analogue of vitamin E, was used as an antioxidant standard. R-Phycoerythrin (R-PE) and ABAP (2,2′-azobis (2-methylpropionamidine) dihydrochloride) were obtained from Sigma Aldrich. Stock solutions of Trolox (6 mM), R-PE (3 × 10^−10^ M) and ABAP (160 mM) were prepared in 75 mM phosphate buffer, pH 7.0. Stock solutions of the plant extracts were prepared in DMSO at 10 mg/mL and subsequently diluted in 75 mM phosphate buffer, pH 7.0. Briefly, 50 μL of each standard solution (five concentrations between 0.4 and 400 μM) or diluted plant extracts (10–100 μg/mL) were mixed with 100 μL R-PE (3 × 10^−10^ M) in a black 96-well microplate and incubated at 37 °C for 5 min before addition of 50 μL 80 mM ABAP. To monitor the ﬂuorescence change over time, the samples were measured at 495 nm (ex)/575 nm (em) every fifth min for 120 min in a spectrofluorometer (SpectraMax iD3, Molecular Devices). Dependent on the antioxidant activity of the plant extract, degradation of the fluorescent protein R-PE by the oxidizing agent ABAP is delayed. When the antioxidants in the sample are depleted, R-PE begins to be degraded by ABPA, leading to a detectable decrease in fluorescence. The antioxidant potential of the three plant extracts was calculated by measuring the lag phase at different concentrations and by calculating the Trolox equivalents using the standard curve. The results are expressed as μM Trolox equivalents (TE) per mg dry extract.

### 2.5 Cell culture

The metastatic human melanoma cell line (A2058) and the epidermal human carcinoma cell line (A431) were obtained from American Type Culture Collection (ATCC, Manassas, VA, USA). A2058 cells were cultured in RPMI-1640 with l-Glutamine supplemented with 10% Fetal Bovine Serum (FBS) and 1% penicillin/streptomycin (Gibco, Thermo Fischer Scientific, Waltham, MA). A431 cells were cultured in Eagle's Minimum Essential Medium (Sigma-Aldrich, Missouri, USA) with 2 mM glutamine, 1% non-essential amino acids and 10% FBS. Primary human epidermal keratinocytes isolated from neonatal foreskin (HEKn, Thermo Fischer Scientific) were maintained in EpiLife growth medium with 60 μM calcium chloride supplemented with 1% of human keratinocyte growth supplement (HKGS) and 0.2% gentamycin/amphotericin (Thermo Fischer Scientific). Cells were cultured at 37 °C in 5% CO_2_ in a humidified atmosphere. All cells were passaged with trypsin-EDTA (0.05%) at 80% confluency.

#### 2.5.1 Cell stimulation

The A2058 cell line was seeded in 12-well plates at a cell density of 10 × 10^4^ cells/well. After 24 h at 37 °C in 5% CO_2_, the culture medium was exchanged for a new medium supplemented with different concentrations of plant extract (15.6, 31.3, 62.5, and 125 μg/mL) and incubated for 2 h at 37 °C in 5% CO_2._ The cells were then further incubated for an additional 46 h with or without 1 μg/mL of lipopolysaccharide (LPS) from *Escherichia coli* serotype 0111:B4 (Sigma-Aldrich). After 48 h, the culture medium was collected and centrifuged for 5 min at 13 000×*g*, and aliquots were immediately frozen at −80 °C, awaiting cytokine quantification.

The A431 and HEKn cell lines were seeded in 12-well plates at a cell density of 10 × 10^4^ cells/well for 24 h. Then, the cells were incubated with different concentrations of plant extracts (15.6, 31.3, 62.5, and 125 μg/mL) for 2 h, and then further incubated for an additional 46 h with or without 10 μg/mL recombinant IFN-γ (Sigma-Aldrich). The supernatants were collected after 48 h and centrifuged for 5 min at 13 000×*g*, and aliquots were immediately frozen at −80 °C, awaiting cytokine quantification.

### 2.6 Cytokine quantification

The extracellular release of IL-6 was determined in the cell culture supernatants using sandwich ELISA. Antibodies and standards were purchased as a Human IL-6 DuoSet ELISA (DY206) from R&D Systems (BioTechne), and the assay were carried out according to manufacturer recommendations. Flat-bottom 96-well immunoplates (MaxiSorp, Nunc) were coated overnight with monoclonal anti-human IL-6 capture antibody. After coating, the plate was blocked with 1% bovine serum albumin (BSA) in phosphate-buffered saline (PBS) for 1 h. After blocking, culture medium and standards (recombinant human IL-6) were added to the plate in duplicates and incubated for 2 h. Biotinylated polyclonal goat anti-human IL-6 detection antibody was then added to the plate. After 2 h, streptavidin labelled HRP was added to the plate and incubated for a further 20 min. Between each incubation step, the plate was washed with PBS with 0.05% Tween20. Substrate (H_2_O_2_ and tetramethylbenzidine 1:1) was added; and after 20 min, the reaction was stopped with 2 N H_2_SO_4_. Optical density was determined with a microplate reader (Molecular Devices SpectraMax D3) at 450 nm with wavelength correction at 570 nm. Quantification of IL-6 in the samples was done from the standard curve with a four parameter logistic curve-fit. The detection limit of the assay was 9.4 pg/mL.

### 2.7 Statistics

Results are expressed as mean ± standard deviation (SD). Data were assessed by two-way ANOVA followed by Dunnett's test using the GraphPad Prism, version 9, statistical software (GraphPad Inc., San Diego, CA, USA). Differences were considered as statistically significant when P was less than 0.05 (P < 0.05); *P ≤ 0.05 **P ≤ 0.01 ***P ≤ 0.001.

## 3 Results

### 3.1 Antioxidant activity and flavonoid content

**Fig. 1:**
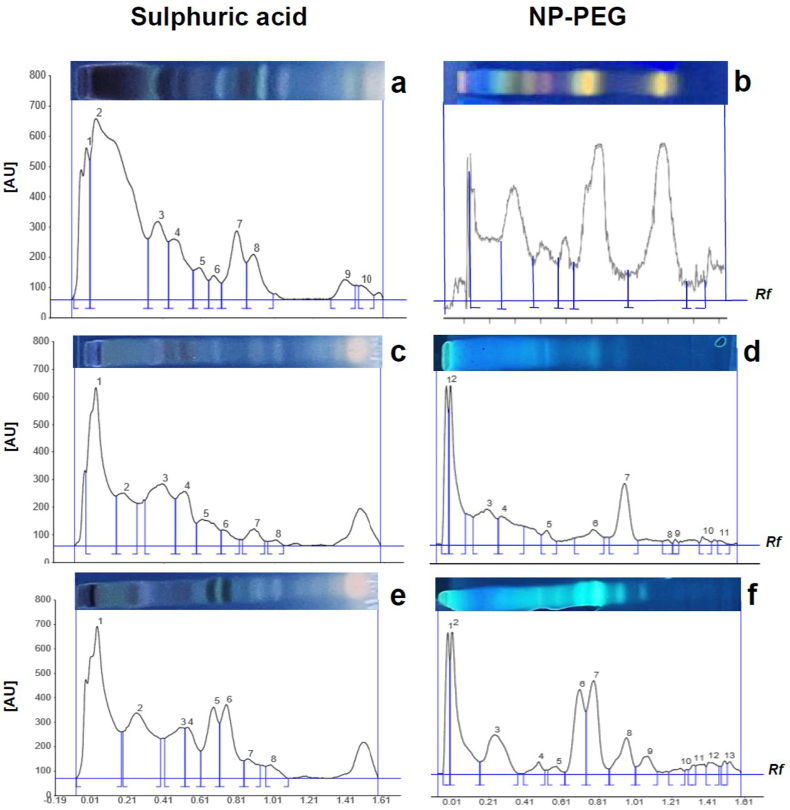
HPTLC analyses where sulphuric acid (a, c, e) and NP-PEG (b, d, f) were used for derivatization visualized and scanned at 366 nm. Densitograms for *R. rosea* (a, b), *P. major* (c, d) and *S. marianum* (e, f) undifferentiated plant callus extracts. Rf; Retention factor (migration distance), AU; Arbitrary Unit (signal intensity).

HPTLC analysis demonstrated that all three extracts contained a complex phytochemical profile with multiple metabolic components. Sulphuric acid derivatization was used to visualize the overall metabolic profile (Fig. 1a, c, 1e), whereas NP-PEG derivatization was used to visualize phenolics and flavonoids (Fig. 1b, d, 1f). The antioxidant capacity in μM Trolox equivalents (TE) and total flavonoid content in quercetin equivalents (QE) of all three extracts are shown in Table 1. A correlation (Pearson's r = 0.94) between the antioxidant capacity and the total flavonoid content in extracts from *P. major*, *R. rosea* and *S. marianum* was observed. Extracts from *S. marianum* had the highest content of flavonoids as well the highest antioxidant activity of the three extracts tested (Table 1). The DMSO amounts used to dissolve the plant extracts did not interfere with the TEAC assay.

**Table 1:** Antioxidant activity in μM Trolox equivalents (TE) per mg dry extract and total flavonoid content expressed as mg quercetin equivalents (QE) per g dry extract. Data shown as mean ± SD (n = 3).

	Antioxidant activity (μM TE/mg)	Total flavonoid content (mg QE/g)
*Silybum marianum* L	0.95 ± 0.07	158.38 ± 2.22
*Plantago major* L	0.42 ± 0.04	29.90 ± 0.90
*Rhodiola rosea* L	0.06 ± 0.006	18.38 ± 0.50

### 3.2 Effect of undifferentiated callus extracts on IFN-γ induced IL-6 release in normal and malignant keratinocytes

**Fig. 2:**
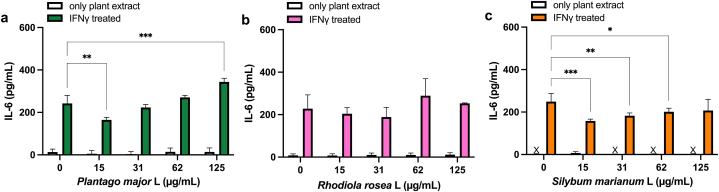
Effect of undifferentiated callus extracts on IFN-γ induced IL-6 production in the epidermal human carcinoma cell line A431. Cells treated with *P. major* (a), *R. rosea* (b), *S. marianum* (c) for 48 h. Data shown as mean ± SD for three independent experiments. x = below detection limit. *P > 0.05, **P < 0.01 or ***P < 0.001 compared to IFN-γ treatment alone.

**Fig. 3:**
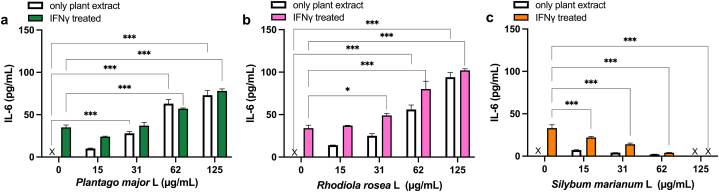
Effect of undifferentiated callus extracts on IFN-γ induced IL-6 production in primary keratinocytes (HEKn). Cells treated with *P. major* (a), *R. rosea* (b), *S. marianum* (c) for 48 h. Data shown as mean ± SD for three independent experiments. x = below detection limit. Significant change to controls is indicated with *P > 0.05, or ***P < 0.001.

First, an inflammatory response of IL-6 release from the cells was induced with IFN-γ. Interestingly, compared with the IL-6 release detected in the cell line A431, IFN-γ induced lower production of IL-6 in HEKn cells (seen in Fig. 2, Fig. 3). The ability of undifferentiated callus extracts from *P. major*, *R. rosea* and *S. marianum* to downregulate *in vitro-*induced IL-6 was then evaluated. The concentrations of the extracts that were selected were considered to be optimal in a dose- and time-dependent manner at concentrations where no or limited impact on the cell viability could be observed. In HEKn and A431 cells, a significant reduction of IFN-γ induced IL-6 release was determined after pre-treatment with *S. marianum* in concentrations ranging between 15 and 125 μg/mL (Fig. 2, Fig. 3c). High concentrations of *P. major* caused a significant increase (P = 0.009) in the IFN-γ induced IL-6 release (Fig. 2a), whereas a significant reduction (P = 0.006) of IL-6 induced release could be observed after pre-treatment with low concentrations of *P. major* in the A431 cell line (Fig. 2a). Extracts from *R. rosea* had no significant effect on IFN-γ induced IL-6 secretion in the A431 cell line (Fig. 2b). Treatment with *P. major* and *R. rosea* in the absence of IFN-γ induced a concentration-dependent increase in the IL-6 production in HEKn (Fig. 3a and b) cells, which was particularity not observed in the other cells used in this study.

### 3.3 Effect of undifferentiated callus extracts on IL-6 release from LPS-treated melanoma cells

**Fig. 4:**
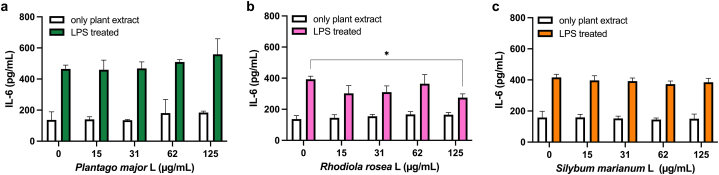
Effect of undifferentiated callus extracts on LPS induced IL-6 production in the melanoma cell line A2058. Cells treated with P. major (a), R. rosea (b), S. marianum (c) for 48 h. Data shown as mean ± SD for three independent experiments. Significant change to LPS induced IL-6 is indicated with *P > 0.05.

An increase of IL-6 secretion was observed in the melanoma cell line A2058 after stimulation with 1 μg/ml LPS (Fig. 4). None of the three tested plant extracts – *P. major*, *R. rosea* and *S. marianum* – had significant effect on LPS-induced IL-6 production in the melanoma cell line A2058 (Fig. 4a–c), with the exception of pre-treatment with a high concentration of the *R. rosea* extract (125 μg/mL), which resulted in a limited but significant (P = 0.01) reduction of IL-6 production (Fig. 4b).

## 4 Discussion

For treatment of cancer, a vast amount of different flavonoids have been studied [32]. Much of the *in vitro*-related work on cancer cells has focused on direct or indirect actions of flavonoids and generated a multitude of anti-cancer effects, such as cell growth and kinase activity inhibition, apoptosis induction, suppression of the secretion of matrix metalloproteinases and of tumour invasive behaviour [15,33,34]. In addition, other reports focus on *in vivo* angiogenesis by dietary flavonoids, or anti-tumour activity in experimental animal studies.

In the present study, we examined the effect of plant extracts from undifferentiated callus cell lines of greater plantain, roseroot and milk thistle and analyzed the antioxidant and anti-inflammatory properties. It is widely agreed that many of the biological effects of plant extracts can be attributed to the antioxidant properties of flavonoids [35,36]. Most investigations have been conducted with individual flavonoids to get a better understanding of the mechanisms of the specific anti-cancer activity [37]. However, final effects of these compounds would be largely influenced by the interactions between the compounds on the same target. This may be one reason as to why the exact mechanism responsible for the antitumor effect of flavonoids is still not thoroughly understood.

In our study, the undifferentiated plant callus extracts from *S. marianum* had a higher total flavonoid content and more vigorous antioxidant activity than the extracts from *P. major* and *R. rosea.* Silibinin, which is the main active component of silymarin, *i.e*., a multicomponent extract from *S. marianum*, has been shown to act as a photodamage sensor that protects or enhances apoptosis in human immortalized keratinocytes (HaCaT), depending on the extent of damage [38]. Studies in a mouse skin SCC model have shown that topical (9 mg in 200 μL acetone) and dietary treatment (50 mg/kg body weight) using Silibinin protect against UVB-induced SCC and BCC formation [28,39,40].

Not only do flavonoids possess antioxidant and antitumor properties, but they are also promising anti-inflammatory compounds. A previous study of a phorbol myristate acetate (PMA)-stimulated THP-1 monocytic cell line showed the ability of P. major methanol extracts to increase the pro-inflammatory cytokine IL-6 at low concentrations (10–25 μg/mL); but at high concentrations (100 μg/mL), the IL-6 production was inhibited [41]. Moreover, Chiang et al. [42], reported that *P. major* hot water extracts induced IFN-γ at low concentrations (<50 μg/mL) in human peripheral blood mononuclear cells (PBMCs). However, these effects were inhibited at high concentrations (>50 μg/mL). Therefore, we investigated if *P. major* was able to modulate LPS and IFN-γ induced IL-6 release from primary keratinocytes and skin cancer cell lines. In the present study different stimuli was used to induce an inflammatory response in the cultured skin cancer cell lines A2058 and A431, and normal primary keratinocytes. Thereafter, the possible suppression of induced IL-6 secretion was measured from the cells. The melanoma cell line A2058 was induced with LPS. Toll-like receptor-4 (TLR4) is neither constitutively expressed nor functional in primary human keratinocytes from neonatal foreskin [43], therefore LPS cannot be used to induce IL-6 secretion in those cells. Since LPS did not trigger IL-6 release in the keratinocytes, IFN-γ was used instead. IL-6 is a multifactorial cytokine that plays a role in both pro-inflammatory and anti-inflammatory pathways [44]. Treatment with a 15 μg/mL *P. major* extract significantly reduced the release of IL-6 in the A431 cell line (P = 0.006). Interestingly, no effects on the release of IL-6 from primary keratinocytes or the melanoma A2058 cell line could be observed. In contrast, treatment of the A431 cell line with a 125 μg/mL *P. major* extract significantly increased IL-6 secretion (P = 0.009). Moreover, treatment with extracts from *S. marianum* significantly reduced IL-6 secretion in both primary keratinocytes and the A431 cell line in all extract concentrations tested (15–125 μg/mL). In the primary keratinocyte cultures, a complete inhibition of IL-6 release induced by IFN-γ was observed after pre-treatment with *S. marianum* at concentrations exceeding 60 μg/mL. This was not observed in the A431 cell line using the same treatment. Not many studies have investigated the effects of silymarin flavonoids on keratinocytes, but Frankova et al. [45] recently showed that induced levels of IL-6 on normal adult human epidermal keratinocytes could be reduced with the addition of different silymarin flavonoids extracted from *S. marianum*. This is in line with the results presented here.

No inhibitory effect of IFN-γ induced IL-6 of *P. major* or *R. rosea* extract could be detected in this study. Contrariwise, extracts of *P. major* and *R. rosea* provoked a concentration-dependent IL-6 response under stressed and non-stressed conditions in normal keratinocytes HEKn, but not in the malignant keratinocytes A431 in this study. Clearly, measuring the release of only IL-6 at one time point will not be sufficient to understand the mode of action or full potential of plant extracts in the context of immunomodulation.

## 5 Conclusion

In conclusion, this study has demonstrated that ethanol extracts of an undifferentiated callus cell line of *S. marianum* possess antioxidant and anti-inflammatory activities in both normal and malignant keratinocytes. Our results clearly suggest that undifferentiated callus extracts of *S. marianum* comprise compounds with a potential to inhibit pro-inflammatory cytokine production in skin cells. Hence, identification of these active compounds needs to be determined. To allow rational design of more potent molecules for the eventual use as possible skin cancer preventive and therapeutic agents, elucidation of the precise mechanisms of any cytotoxicity and downregulation mechanisms of IL-6 secretion is essential.

## Author contribution statement

Anette Gjörloff Wingren, Anna Holefors: Conceived and designed the experiments; Analyzed and interpreted the data; Contributed reagents, materials, analysis tools or data; Wrote the paper.

Riyam Ziyad Faik, Edina Filecovic: Performed the experiments.

Anna Gustafsson: Conceived and designed the experiments; Performed the experiments; Analyzed and interpreted the data; Contributed reagents, materials, analysis tools or data; Wrote the paper.

## Funding statement

This work was supported by the Knowledge Foundation {20170058} {20190010} and the Royal Physiographic Society of Lund, and Biofilms Research Center for Biointerfaces Malmö University, Sweden.

## Data availability statement

Data will be made available on request.

## Declaration of competing interest

The authors declare that they have no known competing financial interests or personal relationships that could have appeared to influence the work reported in this paper.
